# Appreciated

**Published:** 1868-09

**Authors:** 


### APPRECIATED.

For any laudable effort, *success* is the most satisfactory award; but it is human nature to be gratified and encouraged by the appreciation and sympathy of friends during the struggle. Accordingly, we enjoy the following preamble and resolution adopted by the New York Dental Soeiety without dissent, as we are informed, at a meeting in July:—

Whereas, the Ohio Dental College has adopted the practice pursued by literary schools, of classifying its pupils as juniors and seniors, the juniors being students of the first year, and of special branches, and

Whereas, the knowledge that he can not enter the senior class until examined in those branches and found competent, has a tendency to stimulate the junior to greater exertion during his first year, and

Whereas, the division of studies adopted by that school can not but lessen the confusion caused by attempting to perfect a knowledge of a considerable number of studies at the same time, therefore

*Resolved*, That we most heartily commend the plan, and congratulate the Ohio school for their enterprise in inaugurating so excellent a system.

